# Dosage sensitivity of X-linked genes in human embryonic single cells

**DOI:** 10.1186/s12864-019-5432-8

**Published:** 2019-01-14

**Authors:** Jian-Rong Yang, Xiaoshu Chen

**Affiliations:** 1 Department of Medical Genetics, Zhongshan School of Medicine, Sun Yat-sen University, 1212 Medical Science and Technology Building, 74 Zhongshan 2nd Road, Guangzhou, 510080 Guangdong China; 2 Department of Biology, Zhongshan School of Medicine, Sun Yat-sen University, 1227 Medical Science and Technology Building, 74 Zhongshan 2nd Road, Guangzhou, 510080 Guangdong China; 3 RNA Biomedical Institute, Sun Yat-sen Memorial Hospital, Sun Yat-sen University, Guangzhou, 510120 China; 4 Program in Cancer Research, The Fifth Affiliated Hospital, Zhongshan School of Medicine, Sun Yat-sen University, Guangzhou, 510080 China

**Keywords:** Dosage compensation, Sex chromosome, Evolution

## Abstract

**Background:**

During the evolution of mammalian sex chromosomes, the degeneration of Y-linked homologs has led to a dosage imbalance between X-linked and autosomal genes. The evolutionary resolution to such dosage imbalance, as hypothesized by Susumu Ohno fifty years ago, should be doubling the expression of X-linked genes. Recent studies have nevertheless shown that the X to autosome expression ratio equals ~ 1 in haploid human parthenogenetic embryonic stem (pES) cells and ~ 0.5 in diploid pES cells, suggesting no doubled expression for X-linked genes and refuting Ohno’s hypothesis.

**Results:**

Here, by reanalyzing an RNA-seq-based single-cell transcriptome dataset of human embryos, we found that from the 8-cell stage until the time-point just prior to implantation, the expression levels of X-linked genes are not two-fold upregulated in male cells and gradually decrease from two-fold in female cells. Additional analyses of gene expression noise further suggest that the dosage sensitivity of X-linked genes is weaker than that of autosomal genes in differentiated female cells, which contradicts a key assumption in Ohno’s hypothesis, that most X-linked genes are dosage sensitive. Moreover, the dosage-sensitive housekeeping genes are preferentially located on autosomes, implying selection against X-linkage for dosage-sensitive genes.

**Conclusions:**

We observed dosage imbalance between X-linked and autosomal genes, as well as relatively high expression noise from X-linked genes. These results collectively suggest that X-linked genes are less dosage sensitive than autosomal genes, putting one primary assumption of Ohno’s hypothesis in question.

**Electronic supplementary material:**

The online version of this article (10.1186/s12864-019-5432-8) contains supplementary material, which is available to authorized users.

## Background

Mammalian sex chromosomes evolved from a pair of autosomes, in which the evolutionary degeneration of Y potentially caused dosage imbalance between X-linked and autosomal genes. Fifty years ago, Ohno proposed that the expression levels of X-linked genes should be doubled to re-balance the expression dosage between X-linked and autosomal genes [1] in male cells, where only one X chromosome exists. And the doubling of X expression sets stage for the evolution of X-inactivation in female cells, where one of the two X chromosomes becomes transcriptionally inactive [2]. Ohno’s hypothesis formed the theoretical foundation for the current model of mammalian sex chromosome evolution and sex chromosome dosage compensation [3, 4].

In 2006, the first genome-wide empirical test of Ohno’s hypothesis is conducted with a set of microarray-based gene expression profiles in human somatic tissues [5], where the gene expression ratio between one active X and two autosomes (AA) is found as approximately 1, or X:AA ~ 1, lending support to Ohno’s hypothesis [5]. However, gene expression are reflected by probe-specific affinities in microarrays, which perform poorly in quantifying expression ratios [6]. Re-examination of Ohno’s hypothesis using RNA-Seq-based expression profiles [6] found X:AA ~ 0.5, since then the debate over Ohno’s hypothesis in mammals has continued. A number of groups are convinced that Ohno’s hypothesis is correct because the X:AA ~ 1 when only actively expressed genes are considered [7–10]. Accordingly, we replied and emphasized the importance of rigorous calculation and correction for the X:AA ratio [11].

Later on, it was reported that comparison between human X-linked genes and proto-X genes (i.e., the autosomal progenitors of the X-linked genes) suggested no change in per-allele expression levels during mammalian X chromosome evolution [12–15]. Furthermore, the X to autosome expression ratio (X_a_:A) in human parthenogenetic embryonic stem (pES) haploid cells (containing one active X and one set of autosomes) was found to be ~ 1 [5, 16]. Intriguingly, for the X-linked genes encoding components of large protein complexes, which are supposed to be dosage-sensitive, their per-allele expression are upregulated relative to other autosomal members of the same complexes in haploid cells [16], breaking the otherwise balanced dosage, and put the requirement of precise regulation for dosage balance of X-linked genes in question. Collectively, these results have largely refuted the universality of Ohno’s theory in mammals [12]. In contrast, an alternative scenario emerges, i.e., X-linked genes are insensitive to the two-fold expression change caused by evolutionary degeneration of the Y-linked homologs [17]. We speculate that such dosage insensitivity can be further extended to the physiological transition of ploidy (as in meiosis and zygote formation) or X-inactivation during development. In this study, we examined this hypothesis we overarchingly termed as the “insensitive X hypothesis”.

Additionally, we reasoned that a gene with higher dosage sensitivity should display lower expression variance. Similar logic has been invoked in previous studies, in which genes with lower expression variance between individuals are considered under stronger selection on the dosage of expression [18]. Instead of estimating expression variance between biological replicates [18, 19], we took advantage of a recently published single-cell RNA-seq study of human embryos [20] to directly gauge the level of expression noise among individual cells for each gene [21]. This dataset includes the transcriptomes of 1529 individual cells at embryonic days (E) 3–7 from 88 human preimplantation embryos, with a temporal span from the 8-cell stage up to the time-point just prior to implantation [20]. There are a total of 15,633 genes expressed in at least 5 sequenced cells with RPKM (***R***eads ***P***er ***K***ilobase exon model and per ***M***illion mapped reads) no less than 10. This dataset has allowed determination of the sex of each cell by the expression of Y-linked genes and categorization of individual cells into three clearly segregating lineages, namely, trophectoderm (TE), primitive endoderm (PE), and epiblast (EPI) lineages. Analyses of this dataset serendipitously revealed biallelic transcription of *XIST* throughout the progression of X expression dampening, and X-linked genes are transcribed from both alleles in the female preimplantation embryo [20]. This phenomenon is in contrast to the complete silencing of one randomly selected X chromosome in later development [20] (but see [10]).

In testing the insensitive X hypothesis, the single-cell transcriptomic data has at least two advantages. On the one hand, single-cell transcriptome is the state-of-the-art method of cell type categorization, and thus allows estimation of dosage change in the cell subpopulation of different cell types. On the other hand, single cell data shall reveal expression variation among individual cells (of the same cell type), which serves as an approximation for dosage sensitivity of individual genes. Therefore, single-cell transcriptomic data of preimplantation embryos gives us a unique opportunity to test key predictions of the insensitive X hypothesis. First, during the physiological process of X inactivation in female cells, the dosage balance between sex chromosomes and autosomes is expected to change, whereas it should remain unchanged according to Ohno’s theory. Second, the dosage sensitivity, as reflected by diminished expression variation among individual cells, should not be larger for X-linked genes than for autosomal genes. Third, the expression of X-linked genes should be more variable than well-defined dosage-sensitive genes, such as housekeeping genes. In the following sections, we individually test these predictions.

## Results

### Expression levels of X-linked genes are imbalanced with autosomal genes from the early 8-cell stage

As Ohno’s hypothesis concerns genes that existed before the origin of mammalian X, we followed previous studies [6, 17] and focused on human genes with one-to-one orthologs in chicken (Additional file 1: Table S1). For a fair comparison of expression levels, we need to choose unbiased sets of X-linked and autosomal genes. Two strategies were previously employed to that end. On the one hand, a single RPKM limit was used to choose “actively expressed” genes on both X-linked and autosomal genes [7–10]. On the other hand, identical fractions of highly expressed genes were chosen from X and autosomes. That is, if *x*% of X-linked genes and *a*% of autosomal genes were considered expressed by an RPKM threshold, and that *h*% is the smaller of *x*% and *a*%, then *h*% of top highly expressed genes from X, and *h*% of top highly expressed genes from autosomes will be used. Mathematically, the former strategy is only appropriate if X:AA ≈ 1, but overestimates the ratio when X:AA < 1. The latter strategy, however, gives unbiased estimation of X:AA regardless the real ratio [11]. We thus compared, for each day and each lineage, the fraction of X-linked genes (*x*%) whose mean expression level in all single cells is ≥10 RPKM [20] and the same fraction (*x*%, since it is always < *a*%) of autosomal genes with the highest expression levels [16]. The ratio of median mRNA expression levels between X-linked genes and autosomal genes was then calculated and referred to as the X:AA expression ratio.

**Fig. 1:**
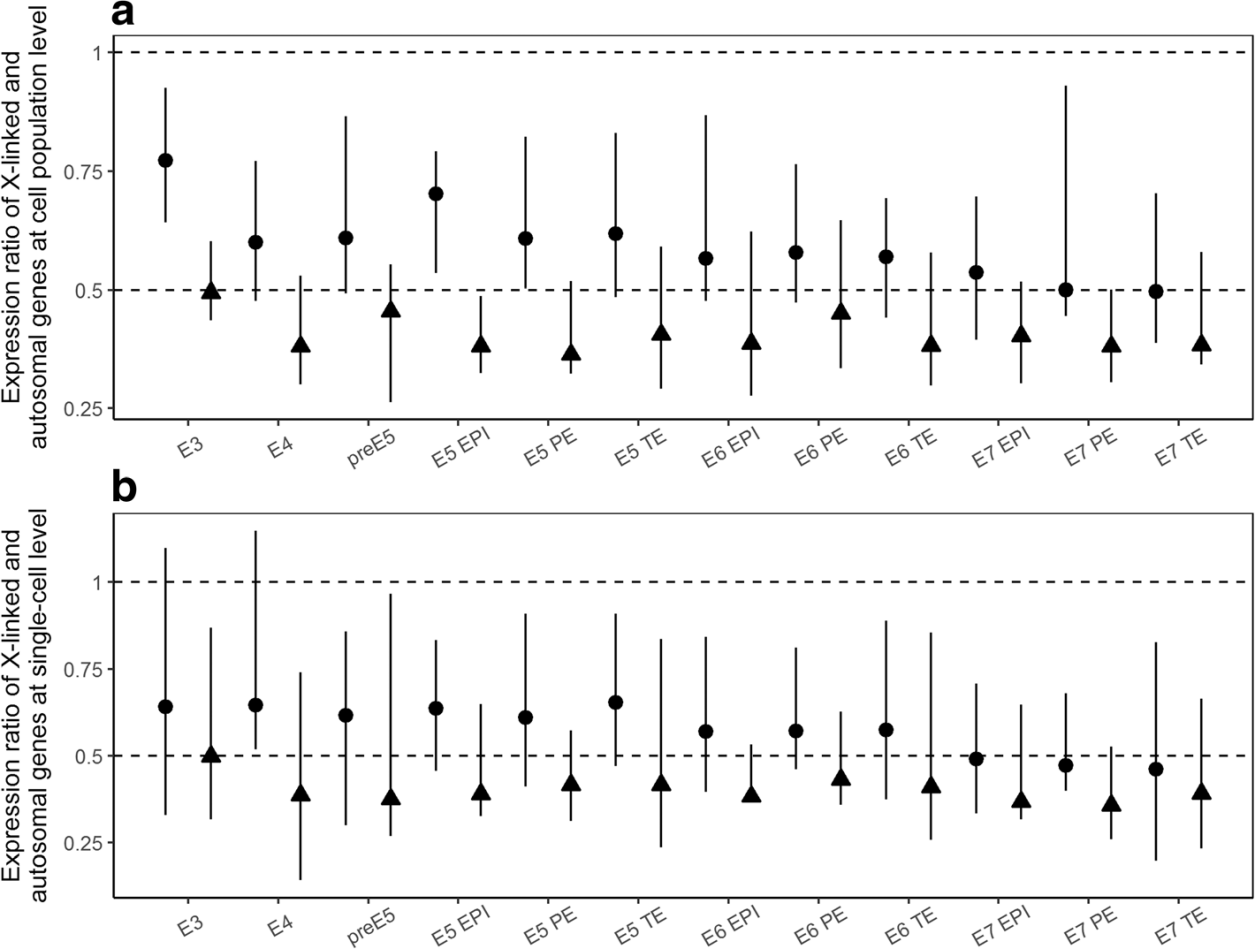
No X-chromosome dosage compensation in human single-cell RNA-seq expression profiles. (**a**) Ratio of the median mRNA expression between X-linked and autosomal genes at cell population level. Error bars show 90% confidence intervals of the medians, estimated by respectively bootstrapping X-linked and autosomal genes 1000 times. (**b**) Ratio of median mRNA expression between X-linked and autosomal genes was calculated for each single-cell. The median and range (minimum to maximum) of these ratios were indicated by the points (circles or triangles) and the error bars, respectively. In all panels, data from male and female cells are represented by triangles and circles, respectively. Two-tailed Mann–Whitney U test was used to test the equality of the mean expression ratio with 1 (filled symbols, *P* < 0.05; open symbols, *P* ≥ 0.05). E3 to E7 indicate embryonic days of the trophectoderm (TE), primitive endoderm (PE) and epiblast (EPI) lineages

We found that the X:AA expression ratio in male cells is ~ 0.5 regardless of lineage and time point (triangles in Fig. 1a). Specifically, the 90% confidence interval of the estimated X:AA expression ratio overlaps with 0.5 but not 1 (Fig. 1a). This result is similar to a previous observation made by RNA-seq in human male diploid cells [6]. On the other hand, the X:AA expression ratio of female cells gradually decreases from ~ 0.75 at E3, to ~ 0.5 at E7 (circles in Fig. 1a). It is also noteworthy that the 90% confidence interval of the X:AA expression ratio of female cells never reaches the prediction made in Ohno’s hypothesis (X:AA = 1). This result is observed in every lineage (Fig. 1a) and remains the same even when we used a less stringent cut-off (RPKM ≥5, Additional file 2: Figure S1a) when filtering expressed X-linked genes, or calculated X:AA separately for each autosome (Additional file 2: Figure S1c). The slightly higher X:AA expression ratio in early time points is likely an intermediate state between haploid cells (X:A ~ 1) and diploid cells (X:AA ~ 0.5) as the maternal to zygotic transition occurs.

To further assess the dosage imbalance between X chromosome and autosomes beyond the cell population-average expression, we computed the X:AA ratio at single-cell level. Following previous report [20], we determined the sex of each cell (Additional file 3: Table S2) based on the expression of Y-linked genes. For each cell, we compared the fraction of X-linked genes whose expression level is ≥10 RPKM [20] and the same fraction of autosomal genes with the highest expression levels in that cell. We then computed the X:AA expression ratio as the ratio of median mRNA expression levels between X-linked genes and autosomal genes in each cell. Similar to the population level results, we found that the X:AA expression ratio is ~ 0.5 in males and gradually decreases to 0.5 in females (Fig. 1b). Moreover, the X:AA expression ratio is always below 1 after E4 in all male and female cells, suggesting that the dosage of expression between X chromosome and autosomes is imbalanced in all cells from E5 onward (Fig. 1b).

It is also important to note that the decrease of expression ratio from oocytic X:A ~ 1 to zygotic X:AA ~ 0.5 occurs early, i.e., before the 8-cell stage, and is quickly finished, such that the X:AA expression ratio reaches ~ 0.5 in no more than a week. If the maintenance of dosage balance between X and autosome is crucial, such fast change of X:AA might be catastrophic to the cellular homeostasis. Together with previous observations [6, 16], these results demonstrate an overall lack of X upregulation at the mRNA level in both male and female preimplantation cells, despite the biallelic expression of X-linked genes in female cells during this period, and suggest the overall insensitivity of X-linked genes to change of dosage relative to autosomal genes.

### The expression noise of X-linked genes is higher than that of autosomal genes in differentiated female cells

The physiological decrease of expression ratio from oocytic X:A ~ 1 to zygotic X:AA ~ 0.5 without interference of normal development implies a lack of phenotypic consequence for different X:AA expression ratios, at least in the range of 0.5 to 1. We thus asked whether X-linked genes are less dosage sensitive than autosomal genes, to which the insensitive X hypothesis would answer “yes”, whereas Ohno’s hypothesis would answer “no”, as it assumes dosage sensitivity for most, especially X-linked genes.

**Fig. 2:**
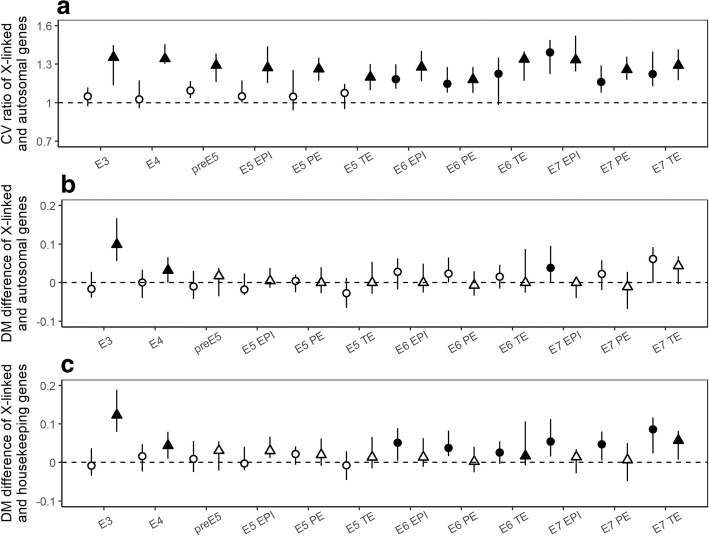
Noisy expression suggest that X-linked genes are less dosage sensitive than expected by Ohno’s hypothesis. **(a)** Ratio of the median CV between X-linked and autosomal genes. **(b)** Difference between the median DM of X-linked and that of autosomal genes. **(c)** Difference between the median DM of X-linked and that of housekeeping genes. In all panels, data from male and female cells are represented by triangles and circles, respectively. Error bars show the 90% confidence intervals of the medians, estimated by respectively bootstrapping X-linked and autosomal or housekeeping genes 1000 times. Two-tailed Mann–Whitney U test was used to test the equality of the CV ratio with 1 (a) or the DM difference with 0 (b and c) (filled symbols, *P* < 0.05; open symbols, *P* ≥ 0.05). E3 to E7 indicate embryonic days of the trophectoderm (TE), primitive endoderm (PE) and epiblast (EPI) lineages. Twenty genes with similar expression levels as the focal gene were used to compute DM

We calculated the ***C***oefficient of ***V***ariation (CV) of mRNA expression for each gene, measured as the standard deviation divided by the mean of single cells with the same lineal status. CV has been considered by some [22] as a direct and unambiguous measure of expression noise (but see below) compared to the expression differences among biological replicates [18]. We then calculated the ratio of the median CV between X-linked and autosomal genes. For males, this X:AA CV ratio is always larger than 1 regardless of lineage and time point (triangles in Fig. 2a). Specifically, the 90% confidence interval of the X:AA CV ratio is always above 1 (Fig. 2a). This result is consistent with previous theoretical predictions of higher expression noise for haploid- than diploid-expressed genes [23, 24]. For females, X-linked genes maintain biallelic expression up to embryonic day 7 [20]. Without the lack of ploidy difference, the X:AA CV ratio is not expected to be higher than 1. We found that in female cells, the CV ratio is slightly higher than 1 from E3 to E5, with the 90% confidence interval overlapping with 1. For female cells on E6 and onward, the CV ratio is significantly higher than 1 (circles in Fig. 2a), which might be caused by lowered expression of female X-linked genes during this time period (Fig. 1). Despite this confounding factor (see below for a better controlled analysis), these findings are consistent with noisier expression of diploid X-linked genes, and therefore lower dosage sensitivity for X-linked genes than autosomal genes.

Because the expression noise represented by CV is potentially confounded by the expression level of the gene, commonly recognized as the finite-number effect [22], we calculated DM, the ***D***istance of its noise (CV) to the ***M***edian noise (CV) of the genes with comparable mean expression levels [21]. Genes with bigger DM are noisier than expected based on the expression level and, therefore, should be less dosage-sensitive. Because DM is defined as the linear distance between the CV of a specific gene and the median CV of genes with similar mean expression levels, DM values should also be compared linearly, i.e., by subtraction, instead of division (as in the case of CV). We found that the median DM of X-linked genes is larger than that of autosomal genes in 5 out of 12 examined male lineages, which was significant in two lineages. In contrast, only two male lineages show the opposite trend, though neither is significant (triangles in Fig. 2b). These observations favor the insensitive X hypothesis over Ohno’s theory, albeit not significantly (5 vs 2 or 2 vs 0). Moreover, haploid genes should theoretically be noisier than diploid genes for similar expression levels [23, 24], but we found no significant increase in DM between X-linked genes and autosomal genes after E4 (Fig. 2b). The slightly higher DM of X compared to AA in early time points is likely a transition state as the maternal to zygotic transition occurs. Combined with the above result on CV (Fig. 2a), these findings suggest that in the male preimplantation cells, the apparent dosage sensitivity of X-linked genes is at least partly due to the finite-number effect, i.e., low expression levels relative to autosomal genes.

On the other hand, in differentiated female lineages from E6 onward, the median DM of X-linked genes is always larger than that of autosomal genes in 6 examined lineages, which is significant for one lineage (circles in Fig. 2b). This pattern, which is supportive of the insensitive X hypothesis, remains qualitatively unchanged when different numbers of genes with comparable mean expression are used to calculate DM (Additional file 4: Figure S2). In addition to the comparison of CV, these findings suggest that at least for differentiated female cells, the insensitive X hypothesis, which is not caused by either the finite-number effect of expression level or the ploidy differences between X and autosomes, is more likely to be true than Ohno’s hypothesis.

Notably, this result is inconsistent with a microarray-based study, which claimed that transcriptional variation of X-linked genes is not different from that of autosomal genes both before and after controlling for transcript abundance [19]. However, this result could be explained by the inability of microarrays to detect variations at the single-cell level and/or small expression differences among genes [6].

### Housekeeping genes exhibit less noise and are preferentially located on autosomes

To further assess the dosage sensitivity of X-linked genes, we compared the DM values of X-linked genes with those of housekeeping genes [25], which are widely considered as dosage-sensitive [21, 26]. By first confirming the reduced expression noise of housekeeping genes (Additional file 5: Figure S3), we compared expression noise of X-linked genes to that of housekeeping genes. We found that the median DM of X-linked genes is larger than that of housekeeping genes in all twelve male cell lineages, among which four are statistically significant (Fig. 2c). This observation is consistent with the expected higher noise of haploid expressed genes [23, 24]. On the other hand, female cells always exhibit significantly noisier expression for X-linked genes than housekeeping genes from E6 onward (Fig. 2c), suggesting that X-linked genes are less dosage-sensitive than housekeeping genes after controlling for the finite-number effect and ploidy differences.

The haploid expression nature and lack of a general mechanism for dosage balancing with autosomal genes make X an undesirable location for dosage-sensitive genes. The insensitive X hypothesis thus also predicts a depletion of housekeeping genes on X. We found that among one-to-one orthologs in chicken, 53 housekeeping genes are located on the X chromosome (out of 360 X-linked genes, Additional file 6: Table S3), which is proportionally less than 2755 out of 11,649 genes on autosomes (*P* = 10^− 4^, Chi-squared test). As housekeeping genes are widely expressed in different tissues, this result is consistent with previous observations that the breadth of expression is lowered for X-linked genes [27, 28].

The depletion of housekeeping genes in X chromosome may have evolved via two scenarios: (i) a chromosome depleted of housekeeping genes becomes a sex chromosome, or (ii) housekeeping genes are removed from the X chromosome once recombination between the therian X and Y is halted. Supporting the latter scenario, a previous study on out-of-X gene movement found that autosomal retrogenes functionally compensate for the silencing of their X-linked housekeeping parental genes [29]. However, dating analyses revealed that retrogenes have been produced since the common ancestor of mammals, whereas the selection for functional compensation driving retrogene export from the X chromosome began much later [29].

Thus, we tested the other scenario, i.e., whether X chromosome has evolved from an autosome depleted of housekeeping genes. Chicken chromosome 1 and 4 consist of regions syntenic to the human X chromosome [30]. We thus respectively compared the fraction of housekeeping genes among all genes with one-to-one orthologs on chicken chromosome 1 and 4 with that on other chicken autosomes. Both chromosome 1 (*P* <  10^− 4^, Chi-squared Test) and 4 (*P* = 0.002, Chi-squared Test) were found to have significantly lower fractions of housekeeping genes than other autosomes (Table 1). This result remains unchanged when only the syntenic region (to human X) of chromosome 1 and 4 are analyzed (Table 1, see Method). The finding that the human X chromosome evolved from autosomes or part of autosomes depleted of housekeeping genes is in line with selective pressure against X-linkage for dosage-sensitive genes. Collectively, our results suggest that X-linked genes are significantly noisier than well-defined dosage-sensitive genes and generally not as dosage sensitive as autosomal genes, which is likely consequence of the evolutionary origin of X from autosomes depleted of housekeeping genes.

**Table 1:** Chromosomes with lower than average numbers of housekeeping genes are predisposed to become sex chromosomes

Chromosome	No. of housekeeping genes with one-to-one orthologs		No. of all genes with one-to-one orthologs		*P* value (Chi-squared Test)
	chicken chr1/4	other chicken autosomes	chicken chr1/4	other chicken autosomes	
Complete chromosomes					
Chicken chr1	316	2384	1615	9840	< 10^−4^
Chicken chr4	160	2540	835	10,620	0.002
Syntenic regions					
Chicken chr1	205	2495	1065	10,390	0.0006
Chicken chr4	149	2551	802	10,653	0.0006

## Discussion

We hereby examined an alternative to Ohno’s hypothesis, i.e., the “insensitive X hypothesis”, where X-linked genes are mostly insensitive to the two-fold expression change caused by either evolutionary degeneration of Y-linked homologs, or the physiological transition of ploidy and X-inactivation during early embryonic development. We utilized recently published single-cell RNA-seq data of human embryos [20] and measured expression noise as a proxy for dosage sensitivity [18]. The biallelic expression [20] of X-linked genes in female cells allows exclusion of noise elevation due to haploid expression [23, 24]. Supporting the “insensitive X hypothesis”, our empirical analysis suggests that X-linked genes are noisier than autosomal genes and are less dosage sensitive than housekeeping genes, at least in the differentiated female preimplantation embryo.

Our study includes some caveats that are worth considering. First, the individual cells were categorized into three clearly segregating lineages (TE, PE and EPI), in which pervasive heterogeneity still exists. However, it is highly unlikely that this source of heterogeneity among single cells influences X chromosomes more than autosomes. Second, instead of directly measuring fitness upon suboptimal expression, dosage sensitivity is inferred from gene expression noise. Although there is evidence for reduced expression noise of genes that are sensitive to dosage [31, 32], fitness effects of gene dosage [33] assessed at the genomic scale would be helpful to further test the insensitive X hypothesis.

How do organisms with incomplete or no dosage compensation avoid deleterious effects of gene dose differences? A previous study in chicken showed that ohnologs, which are duplicated genes known to be dosage-sensitive, are preferentially dosage-compensated on the chicken Z chromosome [34]. As we showed in this study, X-linked genes exhibit noisier expression, and thus, gene-specific dosage compensation may still be suboptimal for X-linked dosage-sensitive genes. Therefore, dosage-sensitive genes are preferentially autosomal, which is achievable by two evolutionary scenarios. One possibility is that dosage-sensitive genes had been removed from the X chromosome [29]. Alternatively, we proposed here that X chromosome has evolved from an ancestral autosome that was depleted of dosage-sensitive genes. This latter scenario is supported by comparison between the human X chromosome and the chicken genome. Because selection-driven gene export from the X chromosome began after the halt of recombination between the therian X and Y [29], evolution of X from chromosomes with fewer dosage-sensitive genes is an evolutionary trajectory with a lower fitness cost for the intermediate genotypes.

In the future, it would be interesting to generate single-cell proteomic data from human cells to validate the above findings at the proteomic level, as was recently carried out for mean protein abundance of a human diploid cell population [35]. It would also be interesting to confirm our results by comparing human haploid transcriptomic or proteomic data with the corresponding data from a bird, as previously reported for diploid transcriptomic data [14, 17].

## Conclusions

Testing the “insensitive X hypothesis” by single-cell transcriptome data of preimplantation human embryos revealed that male X-linked genes are not two-fold upregulated from the 8-cell stage to the time-point just prior to implantation, during which female X-linked genes gradually decrease their expression from oocytic X:A ~ 1 to zygotic X:AA ~ 0.5. Both sexes thus show dosage imbalance between X-linked and autosomal genes. In addition, analyses of expression noise facilitated by single cell data provide novel finding that X-linked genes are not as dosage sensitive as autosomal genes, contrasting the primary assumption of dosage sensitivity for X in Ohno’s hypothesis. Finally, comparative analysis with the chicken genome revealed that X chromosome likely originated from autosomes or part of autosomes that were depleted of housekeeping genes, suggesting selective pressure against X-linkage for dosage-sensitive genes, a new factor potentially constrains the evolutionary origin of sex chromosomes.

## Methods

Gene models and mapping of EnsEMBL gene IDs to UniProt/SwissProt accessions in human were downloaded from EnsEMBL (release 87) [36]. Human and chicken one-to-one orthologs were also downloaded from the same release of EnsEMBL. Syntenic regions of human X chromosome in the chicken chromosome 1 and 4 were previously identified [30] and further constrained here to include only genes lying between the first and last one-to-one ortholog within that region. Genes expressed uniformly across a panel of tissues captured by RNA-seq are identified as human housekeeping genes [25]. The number of RNA-seq *r*eads *p*er *k*ilobase of exon per *m*illion reads mapped (RPKM), as found in the supplementary data of the original study, was downloaded and directly used as gene expression levels [20]. To avoid the effect of technical noise in single-cell expression measurements, especially for lowly expressed genes, we followed previous procedures and focused on X-linked genes with RPKM ≥10 [20]. We also tried a less stringent cut-off (RPKM ≥5), but the results were quantitatively unchanged (Additional file 2: Figure S1a and b). At the cell population level and for each embryonic day and each lineage, we first determined the sex of each cell by the expression of Y-linked genes. Then we compared the fraction of X-linked genes whose expression level was at least 10 or 5 RPKM with the same fraction of autosomal genes that had the highest expression level [16]. The number of genes whose RPKM surpass these expression thresholds (5 or 10) was listed in Additional file 7: Table S4. In addition, for each single cell, we compared the fraction of X-linked genes whose expression level was at least 10 or 5 RPKM with the same fraction of autosomal genes that had the highest expression level. We then computed the ratio of the median mRNA expression level between X-linked genes and autosomal genes. To compare expression noise, we calculated the CV of mRNA expression for each gene at the cell population level, measured as the standard deviation divided by the mean, and computed the ratio of the median CV between X-linked genes and autosomal genes. As another measurement of expression noise, we used DM, which was calculated as previously described [21]. Briefly, we ranked the genes by their mean expression, and then for each specific gene, we used 10, 20 or 50 genes with similar levels of focal gene to calculate the median CV, and the difference between the median CV and the focal CV was used as DM [21]. We then calculated the difference in median DM between X-linked genes and autosomal genes, as well as between X-linked genes and housekeeping genes.

## Additional files

Additional file 1: **Table S1.** List of human genes with one-to-one orthologs in chicken. (XLSX 557 kb)

Additional file 2: **Figure S1.** No X-chromosome dosage compensation in human single-cell RNA-seq profiling. (a and b) Similar to Fig. 1 except that X-linked genes with RPKM no less than 5 are considered. (c) Using X-linked genes with RPKM no less than 10, ratio of the median mRNA expression between X-linked and autosomal genes at the cell population level was calculated for each autosome separately, resulting 22 X:AA ratios for each cell lineage. The median and range (minimum to maximum) of these 22 X:AA ratios were indicated by the points and the error bars, respectively. Triangles and circles are respectively representing data from male and female cells. The distributions always overlaps with X:AA = 0.5, but not X:AA = 1. (TIF 18794 kb)

Additional file 3: **Table S2.** No.of Cells in each sex of each lineage. (XLSX 9 kb)

Additional file 4: **Figure S2.** Noisy expression suggest that X-linked genes are less dosage sensitive than expected by Ohno’s hypothesis. (a and b) Similar to Fig. 2b and c except that 10 genes with similar expression levels as the focal gene are used to compute DM. (c and d) Similar to Fig. 2b and c except that 50 genes with similar expression levels as the focal gene are used to compute DM. (TIF 18382 kb)

Additional file 5: **Figure S3.** Housekeeping genes are more dosage sensitive than other autosomal genes. Similar to Fig. 2b except that the DM of autosomal housekeeping genes is compared to that of autosomal genes. (TIF 4285 kb)

Additional file 6: **Table S3.** List of human housekeeping genes with one-to-one orthologs in chicken. (XLSX 133 kb)

Additional file 7: **Table S4.** Number of genes whose mean RPKM surpass the expression threshold in each cell lineage. (XLSX 15 kb)

## Abbreviations

CV: Coefficient of Variation
DM: the Distance of the noise (CV) to the Median noise (CV)
EPI: Epiblast
PE: Primitive endoderm
pES: Parthenogenetic embryonic stem cells
RPKM: Reads Per Kilobase exon model and per Million mapped reads
TE: Trophectoderm
