# Supplementary figures and images for: Dosage sensitivity of X-linked genes in human embryonic single cells
Source: BMC Genomics. 2019 Jan 14;20:42. doi: 10.1186/s12864-019-5432-8 (PMC6332578; doi:10.1186/s12864-019-5432-8)

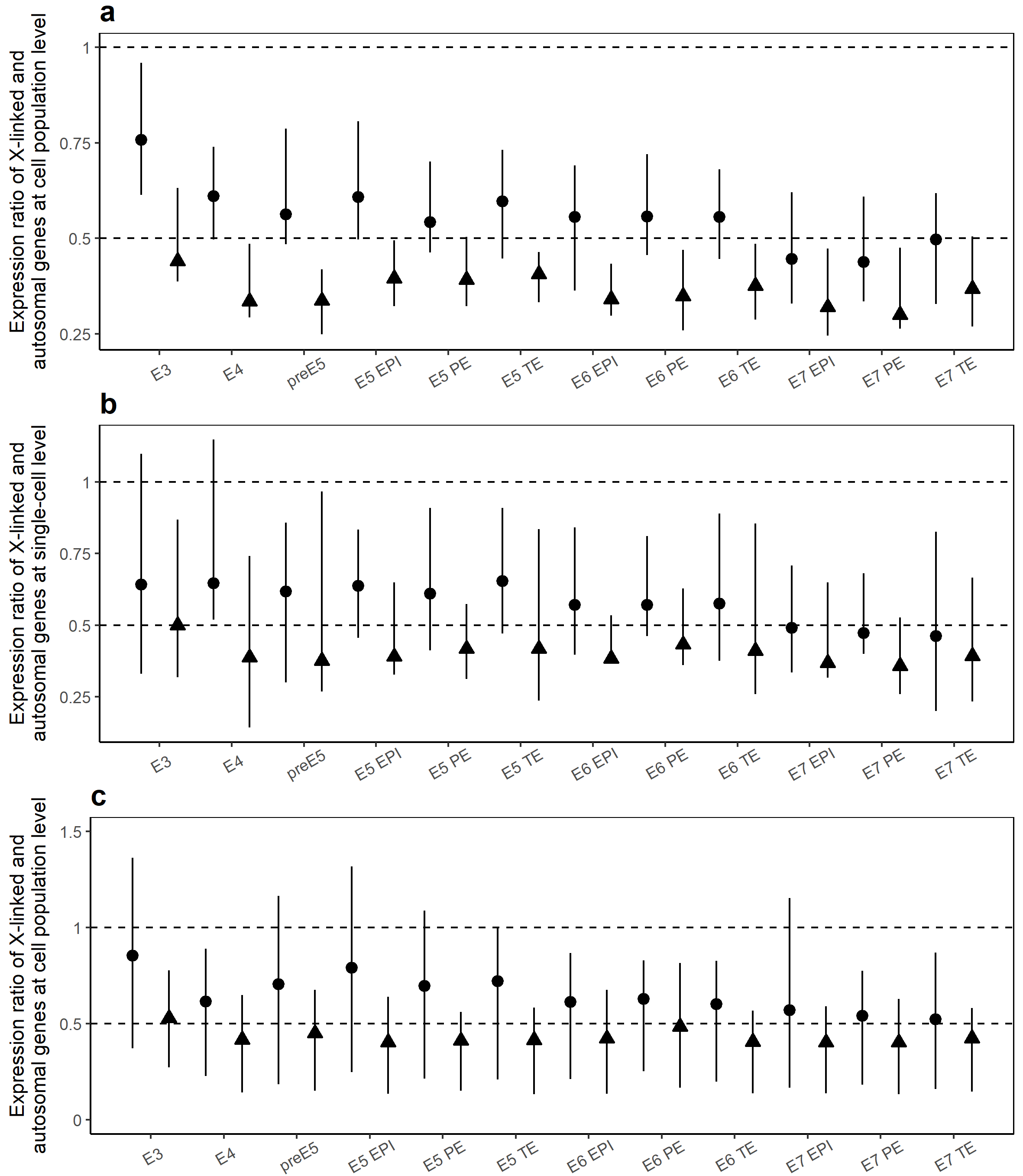

Supplement: Supplementary file 2 — Figure S1. No X-chromosome dosage compensation in human single-cell RNA-seq profiling. (a and b) Similar to Fig. 1 except that X-linked genes with RPKM no less than 5 are considered. (c) Using X-linked genes with RPKM no less than 10, ratio of the median mRNA expression between X-linked and autosomal genes at the cell population level was calculated for each autosome separately, resulting 22 X:AA ratios for each cell lineage. The median and range (minimum to maximum) of these 22 X:AA ratios were indicated by the points and the error bars, respectively. Triangles and circles are respectively representing data from male and female cells. The distributions always overlaps with X:AA = 0.5, but not X:AA = 1. (TIF 18794 kb) [file 12864_2019_5432_MOESM2_ESM.tif]

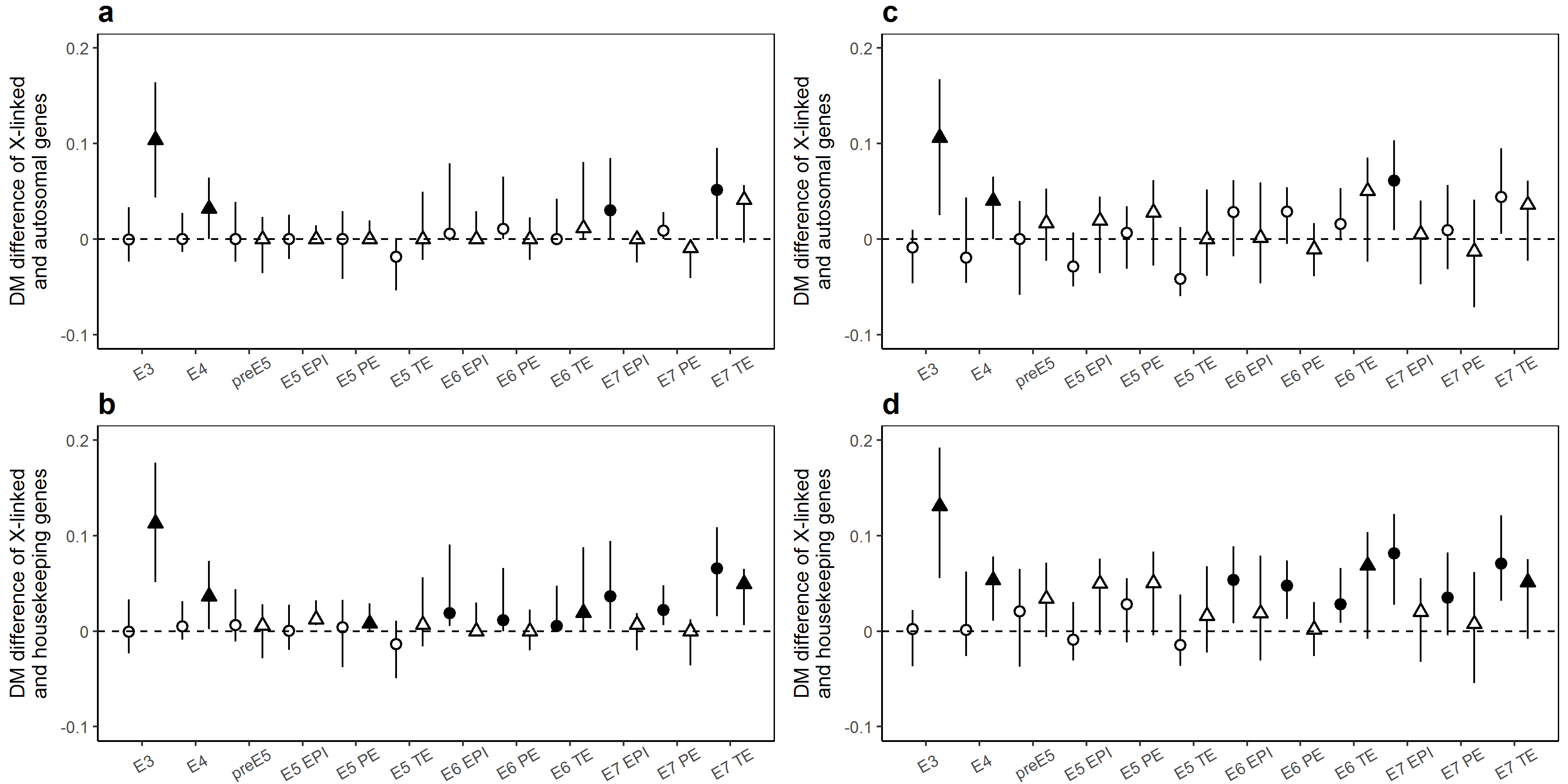

Supplement: Supplementary file 4 — Figure S2. Noisy expression suggest that X-linked genes are less dosage sensitive than expected by Ohno’s hypothesis. (a and b) Similar to Fig. 2b and c except that 10 genes with similar expression levels as the focal gene are used to compute DM. (c and d) Similar to Fig. 2b and c except that 50 genes with similar expression levels as the focal gene are used to compute DM. (TIF 18382 kb) [file 12864_2019_5432_MOESM4_ESM.tif]

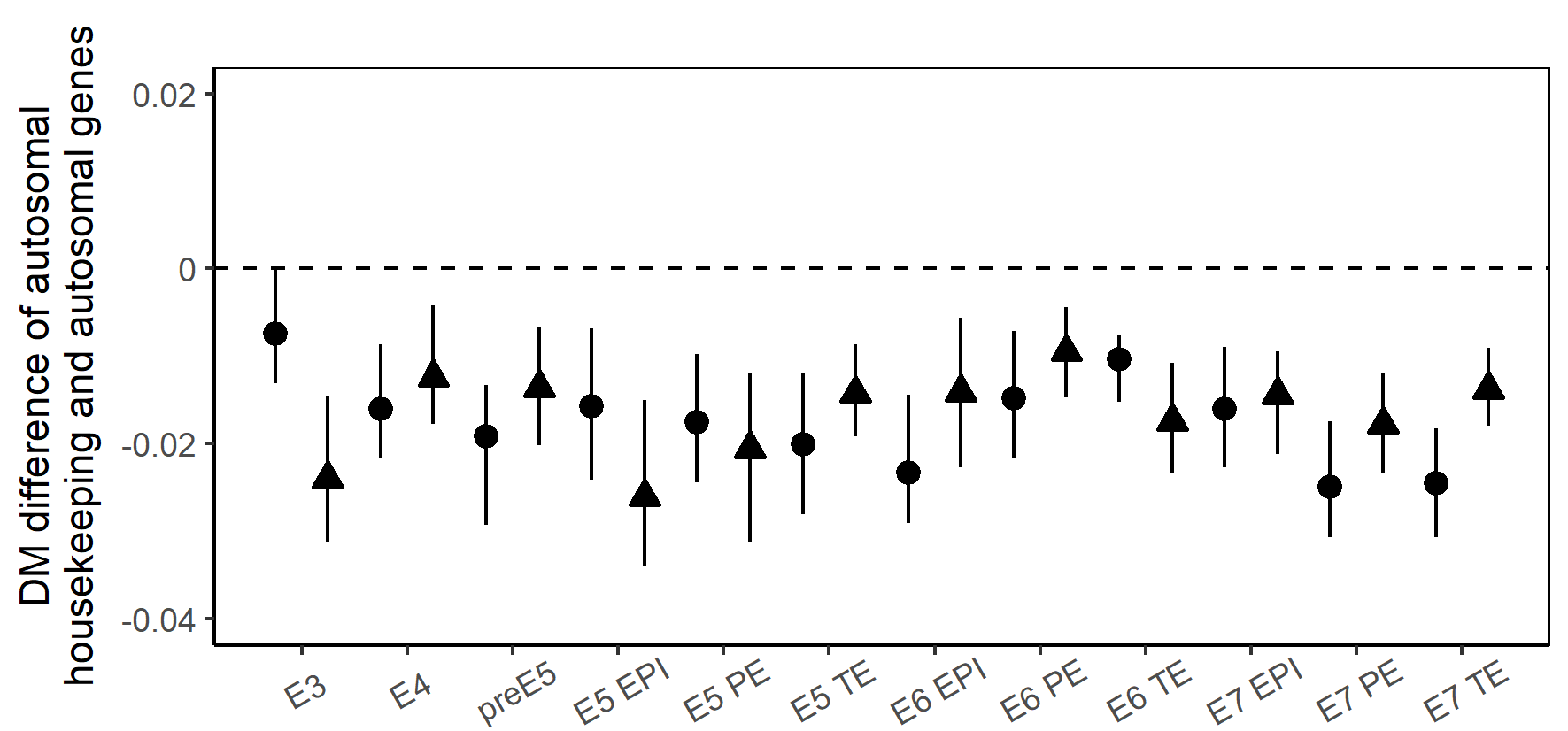

Supplement: Supplementary file 5 — Figure S3. Housekeeping genes are more dosage sensitive than other autosomal genes. Similar to Fig. 2b except that the DM of autosomal housekeeping genes is compared to that of autosomal genes. (TIF 4285 kb) [file 12864_2019_5432_MOESM5_ESM.tif]
